# Structural Effects of Lanthanide Dopants on Alumina

**DOI:** 10.1038/srep39946

**Published:** 2017-01-06

**Authors:** Ketan Patel, Victoria Blair, Justin Douglas, Qilin Dai, Yaohua Liu, Shenqiang Ren, Raymond Brennan

**Affiliations:** 1 Department of Mechanical Engineering and Temple Materials Institute, Temple University, Philadelphia, PA 19122, USA; 2 US Army Research Laboratory, Aberdeen Proving Ground, MD 21005, USA; 3 Molecular Structures Group, University of Kansas, Lawrence, KS 66045, USA; 4 Quantum Condensed Matter Division, Oak Ridge National Laboratory, Oak Ridge, TN 37831, USA.

## Abstract

Lanthanide (Ln^3+^) doping in alumina has shown great promise for stabilizing and promoting desirable phase formation to achieve optimized physical and chemical properties. However, doping alumina with Ln elements is generally accompanied by formation of new phases (i.e. LnAlO_3_, Ln_2_O_3_), and therefore inclusion of Ln-doping mechanisms for phase stabilization of the alumina lattice is indispensable. In this study, Ln-doping (400 ppm) of the alumina lattice crucially delays the onset of phase transformation and enables phase population control, which is achieved without the formation of new phases. The delay in phase transition (θ → α), and alteration of powder morphology, particle dimensions, and composition ratios between α- and θ-alumina phases are studied using a combination of solid state nuclear magnetic resonance, electron microscopy, digital scanning calorimetry, and high resolution X-ray diffraction with refinement fitting. Loading alumina with a sparse concentration of Ln-dopants suggests that the dopants reside in the vacant octahedral locations within the alumina lattice, where complete conversion into the thermodynamically stable α-domain is shown in dysprosium (Dy)- and lutetium (Lu)-doped alumina. This study opens up the potential to control the structure and phase composition of Ln-doped alumina for emerging applications.

Aluminum oxide (Al_2_O_3_), also known as alumina, is a common ceramic material of interest for advanced applications. Apart from the most thermodynamically stable α-phase, alumina also exists in several metastable/transitional phases^1^. These metastable phases are classified into two major categories, including face centered cubic (FCC) and hexagonal closed packing (HCP) anionic arrangements^2^. These polymorphs depend on the arrangement of cations within each oxygen sub-group. The existence of several crystalline polymorphs and vacant octahedral sites enables alumina to be adopted for a variety of doping studies and technological applications^3^,^4^,^5^. Lanthanide (Ln^3+^) ion-doped Al_2_O_3_ is particularly appealing, since the local environment of the doped ions is known to critically influence the optical and mechanical properties of Al_2_O_3_ due to the large size mismatch between Ln dopants and aluminum^6^,^7^,^8^. For example, the presence of dopants (ytterbium, gadolinium or lanthanum) strengthens the grain boundaries of alumina, largely affecting the mechanical properties^9^,^10^,^11^. A study conducted on doping alumina with lanthanum (La) showed a reduction in the phase transformation (γ → α) temperature by 100 °C, thus increasing the thermal stability of γ-Al_2_O_3_^12^. Although Ln doping has shown favorable effects on alumina, understanding the dopant location within the alumina matrix remains a challenge. Due to the large size of the Ln-ions compared to Al^3+^ (0.054 nm for Al^3+^ and 0.103 nm → 0.0861 nm for Ln^3+^ series), which results in an ionic size mismatch between the dopants (Ln^3+^) and the Al^3+^ cations, the solubility of lanthanide cations in alumina is a significant challenge^13^,^14^. Additionally, there is a limited understanding of the local structures and distributions of Ln-ions within the alumina matrix^15^,^16^,^17^,^18^,^19^. Previous reports on Ln-doping of alumina have generally been conducted at doping concentrations of 0.5–5 wt%^20^,^21^,^22^,^23^,^24^,^25^. The formation of a new phase, LnAlO_3_ or Ln_2_O_3_, is observed in previously reported cases, which complicates understanding of the role Ln-doping plays on structure and phase transition of the alumina lattice^26^,^27^. This also makes it intriguing to assess the role of other Ln-elements as “structural promoters” due to their phase transformation delays and resulting effects on the structure of doped alumina.

## Results and Discussion

In this study, the effects of sparse Ln^3+^ dopants [Lanthanum (La), Praseodymium (Pr), Dysprosium (Dy), Neodymium (Nd), Erbium (Er), Thulium (Tm), Lutetium (Lu), Gadolinium (Gd) and Ytterbium (Yb)] on the phase transition and structural geometry of alumina are reported. The concentrations of Ln-dopants were kept at a constant level of Ln_0.002_Al_1.998_O_3_ (e.g. the weight concentration of Er-dopant was ~0.03271 wt% (or 400 ppm) in the alumina lattice) to avoid any potential formation of LnAlO_3_ and/or Ln_2_O_3_ phases. Furthermore, in light of the great importance of Ln-doped alumina and the challenges associated with the analysis of substituent species in crystalline Al_2_O_3_ materials, five possible locations were proposed for the Ln-dopants, and experimental efforts were made to find the most chemically reasonable location. A suite of state-of-the-art structural probes was utilized to test the hypothesis that the local structure and positioning of Ln-ions could largely influence the structural transition and phase composition of doped alumina due to their interactions with the Al_2_O_3_ host lattice. 1D magic angle spinning (MAS) and 2D ^27^Al multiple quantum magic angle spinning (MQ-MAS) solid-state nuclear magnetic resonance (ss-NMR) spectroscopy techniques, together with high resolution X-ray diffraction (Synchrotron 11-BM at the Advanced Photon Source of Argonne National Laboratory), were employed to examine the Ln-dopant effect on Al_2_O_3_ phase composition. The structural characteristics and phase evolution of Ln-doped Al_2_O_3_ were further confirmed through scanning electron microscopy (SEM) and high temperature differential scanning calorimetry (HT-DSC). A wide range of different spectroscopic techniques was employed to enable a detailed understanding of the local structure of Ln^3+^ ions in Al_2_O_3_ and provide critical insight into the rational design of alumina materials.

**Figure 1:**
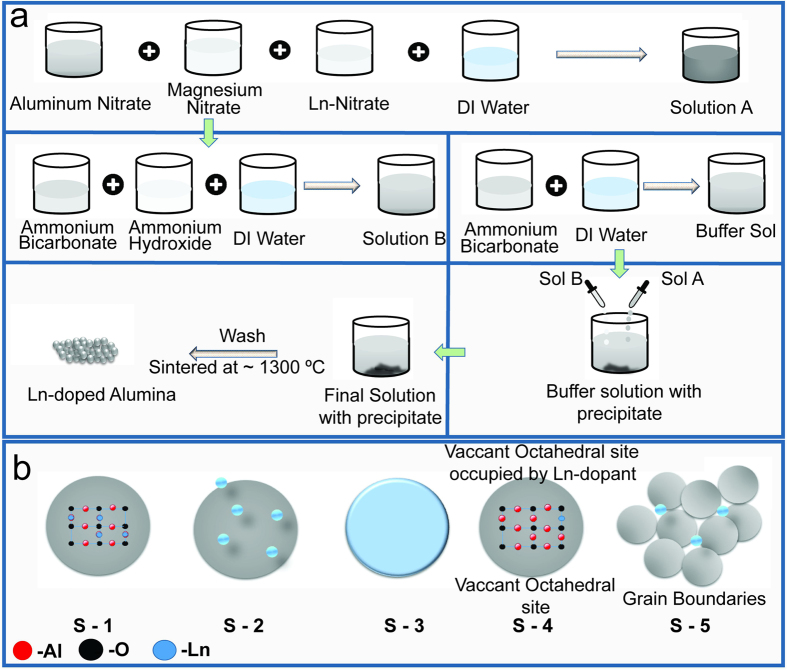
(**a**) Schematic representation of the Ln-doped alumina sample preparation. (**b**) Schematic representation of the five possible locations for the Ln-dopants to reside in alumina. [S-1: - Ln-dopant intercalating in the lattice by substituting some of the aluminum cations in the octahedral sites. S-2: - Formation of LnAlO_3_ or Ln_2_O_3_ nanoparticles, which were entirely separate from the alumina nanoparticles. S-3: - Formation of a layer or a protective shell of LnAlO_3_ or Ln_2_O_3_ around the alumina nanoparticles. S-4: - Ln-dopants residing in the one-third vacant octahedral sites or the vacant tetrahedral sites. S-5: - Ln-dopants residing on the grain boundaries of the alumina lattice].

All of the Ln-doped alumina samples referenced in this study were prepared by mixing an acidic solution of aluminum nitrates, magnesium nitrates, and lanthanide nitrates in stoichiometric amounts (as shown in Fig. 1a, targeting a composition of Ln_0.002_Al_1.998_O_3_ with 250 ppm of Mg^2+^) with a basic solution. The addition of Mg^2+^ to the composition was believed to have an influence over structural distortions that could assist in dissolving Ln^3+^ into the Al^3+^ octahedral sites. There is significant literature detailing the role of MgO additives for enhancing the sintering behavior of alumina, suggesting that the MgO acts as a grain growth inhibitor, leading to a more uniform grain structure^27^,^28^,^29^,^30^. However, these studies examined significantly larger quantities of MgO than are reported here. After calcination of the resulting powder, Ln-doped alumina was obtained and further confirmed using a suite of advanced spectroscopy techniques. In addition, the location of the Ln dopants in alumina has been a topic of debate in the past and is still ongoing^21^,^27^,^28^,^29^,^30^. Five possible locations were identified for the Ln-dopants to reside (Fig. 1b): (i) intercalating in the alumina lattice by substituting some aluminum cations, (ii) creating LnAlO_3_ or Ln_2_O_3_ phases which are entirely separate from the alumina phase, (iii) forming a shell of LnAlO_3_ or Ln_2_O_3_ around the alumina core particles, (iv) residing in the vacant octahedral or tetrahedral sites, or (v) residing on the grain boundaries of the alumina lattice^21^,^31^,^32^.

Substitution of larger atomic radius La atoms into the Al sites of the alumina lattice would have potentially resulted in a larger bond distance of La-O (2.3–2.8 Å) as compared to that of Al-O (1.8 Å). However, such distortions were not reflected in the spectroscopy studies (shown in a later section), and it seemed unlikely that the La atoms were substituted for the Al atoms in the alumina lattice, as suggested in scenario (i). Furthermore, scenarios (ii) and (iii) were ruled out due to the amount of dopant used in this study, as ~0.03271% (by weight) of the total molecular weight of Ln_0.002_Al_1.998_O_3_ was not enough for the formation of separate phases or layers of LnAlO_3_/Ln_2_O_3_. This was also confirmed through spectroscopy studies in this report^8^. In addition, assuming a homogeneous lattice with sparse Ln cations well dispersed in the matrix, the distortion that occurred from one cation would not interfere with other dopants. Since a short and fast calcination schedule was used to process the material, there was not enough time for the cations to migrate towards one another and form a secondary phase.

**Figure 2:**
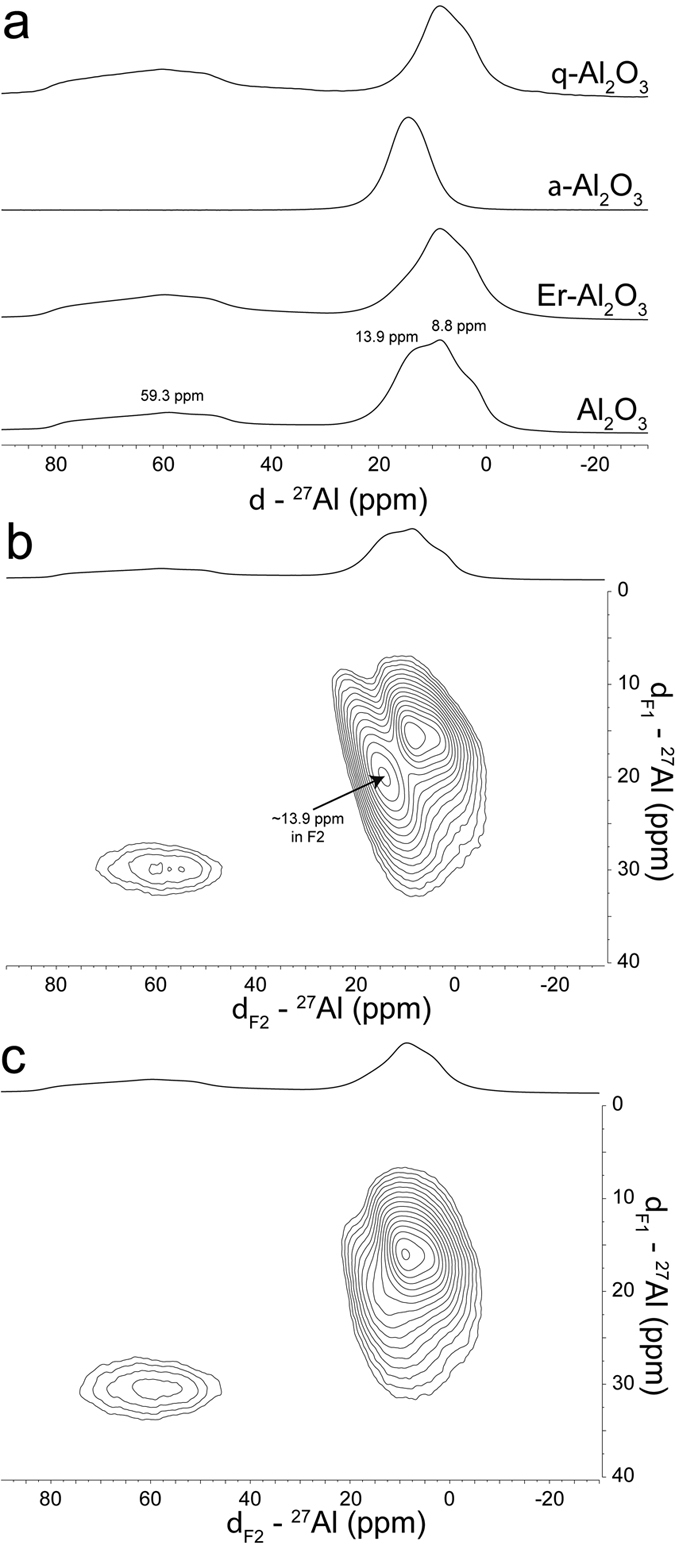
(**a**) Overlay of 1D ^27^Al MAS NMR spectra for Al_2_O_3_, Er-doped Al_2_O_3_, un-doped α-Al_2_O_3_ phase and un-doped θ-Al_2_O_3_ phase. The MAS rate was equal to 10 kHz. (**b**) 2D ^27^Al MQ-MAS spectra of un-doped Al_2_O_3_ (**c**) 2D ^27^Al MQ-MAS spectra of Er-doped Al_2_O_3_.

To understand the location of the Ln-dopants and structural changes developed in Al_2_O_3_, Er-doped alumina was selected as the first prototype doping example. The Al_2_O_3_, Er-doped Al_2_O_3_, pure α-Al_2_O_3_, and pure θ-Al_2_O_3_ samples were characterized using 1D ^27^Al Magic Angle Spinning (MAS) ss-NMR with spin rate of 10 kHz (Fig. 2a). To isolate pure phases of α-Al_2_O_3_ and θ-Al_2_O_3_, the calcination temperature was adjusted such that only α- and θ-Al_2_O_3_ were detected by X-ray diffraction. The ^27^Al chemical shift depended strongly on the number of coordinating oxygens^33^. The α-Al_2_O_3_ was represented by hexagonal packing of oxygen anions. The distribution of Al cations for this phase was limited to octahedral sites, and was represented by a single peak at ~13.9 ppm in the MAS spectrum. The θ-Al_2_O_3_ phase, on the other hand, was represented by monoclinic arrangements of the oxygen and distribution of Al cations in tetrahedral and octahedral sites. This arrangement led to two distinct inhomogeneously broadened peaks at ~59.3 and ~8.8 ppm for tetrahedral and octahedral sites, respectively. Additionally, there was a downfield shoulder at ~2.7 ppm on the peak at ~8.8 ppm for θ-Al_2_O_3_. The 1D ^27^Al MAS ss-NMR spectrum of Al_2_O_3_ represented a weighted summation of the spectra of pure α- and θ- phase Al_2_O_3_. Upon doping Al_2_O_3_ with 400 ppm Er^3+^, the MAS spectrum changed relative to undoped Al_2_O_3_. Most notably, there was significant attenuation, which reduced the intensity of the peak at ~13.9 ppm.

Multi-dimensional ss-NMR was used to further explore these results. Since ^27^Al is a quadrupolar nuclei (S = 5/2), the signals in 1D ^27^Al ss-NMR spectra were broadened by quadrupolar coupling, as well as other anisotropic spin interactions, including chemical shift anisotropy and dipolar coupling^34^,^35^. The MQ-MAS experiment^36^ provided a tool to discern these interactions. This multi-dimensional experiment correlated the spectrum without second order quadrupolar anisotropic effects in the indirect dimension (y-axis, F1) while the spectrum was broadened by these anisotropic effects in the direct dimension (x-axis, F2), making the MQ-MAS experiment useful for resolving overlapping peaks. The 2D ^27^Al MQ-MAS spectra were collected on undoped and Er^3+^-doped Al_2_O_3_ (Fig. 2b and c). There were three clear peaks in the MQ-MAS spectrum of Al_2_O_3_ at ~59.3, 13.9 and 8.8 ppm, respectively, in the F2 dimension. The latter peak included a downfield shoulder at ~2.7 ppm. The intensity of the peak at ~13.9 ppm in F2 in the MQ-MAS spectrum was reduced significantly upon doping with Er^3+^, which was in agreement with the results of the 1D MAS spectra. Taken together, these results suggested that, given the ~0.03271% weight percentage of dopant, changes in the NMR spectra were not attributed to Er^3+^ induced changes to anisotropic spin interactions, but an alteration in alumina phase composition caused by the doping procedure (Fig. 1a).

**Figure 3:**
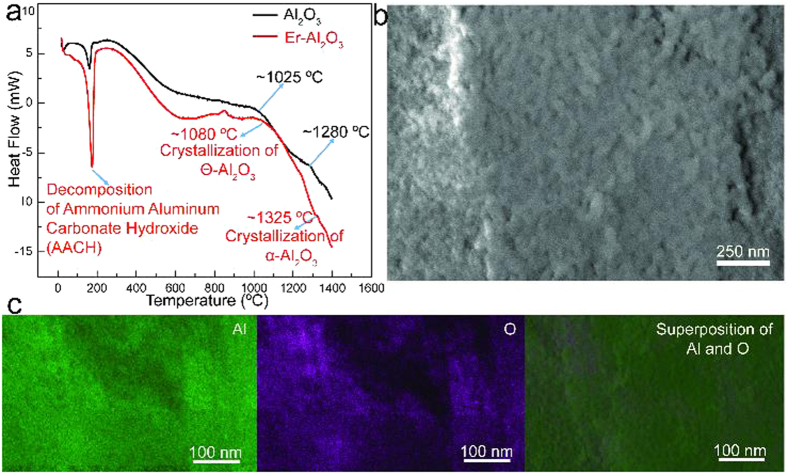
(**a**) High temperature differential scanning calorimetry (HT-DSC) curves of Al_2_O_3_ (purple) and Er-Al_2_O_3_ (red). (**b**) SEM image of Er-doped Al_2_O_3_. (**c**) EDS elemental mapping of Er-Al_2_O_3_.

To reveal the Er-dopant effect on the phase composition and structure of alumina, HT-DSC was utilized to investigate the phase transition of alumina before and after Er-doping. Despite limited solubility, Ln-dopants effectively altered the phase transition temperature of alumina, as more thermal energy was necessary to convert to the α-Al_2_O_3_ phase, the most thermodynamically stable phase, after Er-doping^37^,^38^. The phase transition took place at a temperature (T_c_) of ~1280 °C for the pristine Al_2_O_3_ (Fig. 3a) and ~1325 °C for the Er-doped Al_2_O_3_, suggesting that doping alumina with Er increased the transition temperature significantly. SEM images of Er-doped Al_2_O_3_ (Fig. 3b) calcined at 1300 °C, which was lower than the alpha alumina transition temperature for Er-doped Al_2_O_3_, showed a mixture of particle morphologies. Theta alumina has been known to have a smaller particle size than alpha alumina, as the grains will not grow until the final alpha alumina phase has been developed. It has been theorized that the larger grains represent alpha alumina and the smaller grains represent theta alumina. These observations further confirmed the NMR spectra of Er-doped Al_2_O_3_ discussed above. Loading of large Er^3+^ ions into alumina forced the lattice to expand, distorting the neighboring tetrahedral and octahedral locations. Consequently, the distorted lattice conversion into octahedral sites and rearrangement into stable structures for the phase transformation from θ-Al_2_O_3_ to α-Al_2_O_3_ required a higher thermal energy, leading to a phase transformation temperature shift.

**Figure 4:**
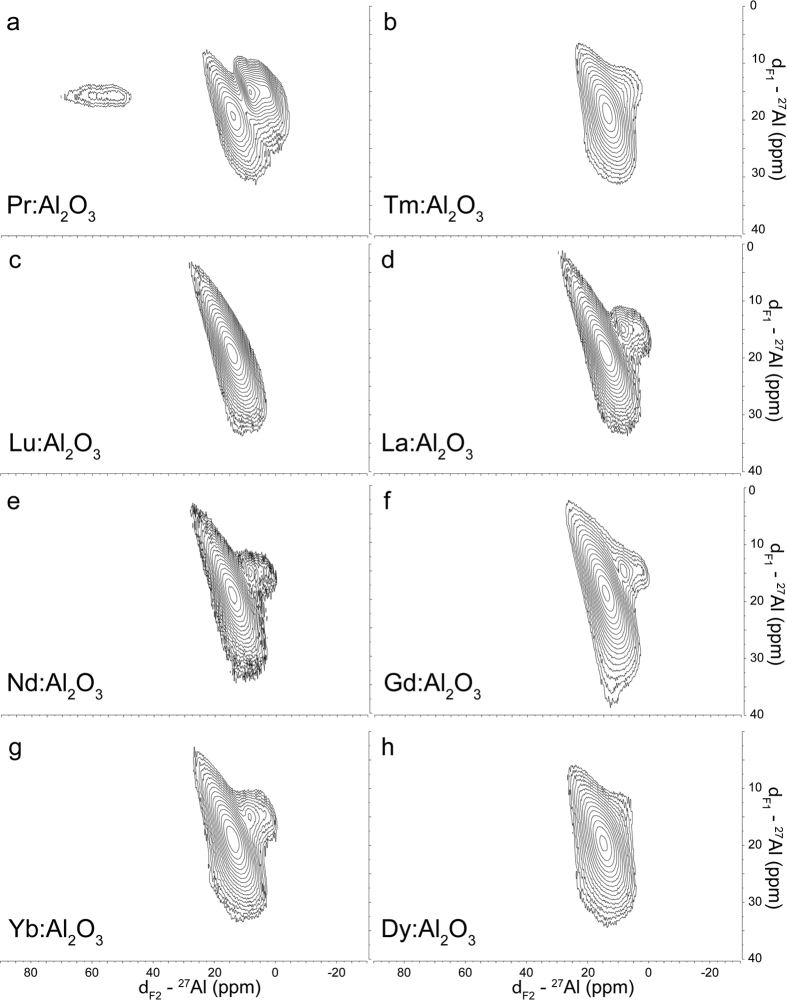
The 2D MQ-MAS spectra of Ln-doped Al_2_O_3._

In order to confirm the hypothesis that Ln-dopants altered the phase transition temperature in alumina, and to distinguish the doping mechanisms between scenarios (iv) and (v), eight additional lanthanide elements were selected to gain further understanding of the Ln dopant effects. Dopants, including La, Pr, Dy, Nd, Tm, Lu, Gd and Yb, were chosen with the same doping levels as the Er-dopant, and their domain structures were characterized using ss-NMR. 2D MQ-MAS spectra (MAS rate of 12 kHz) of Pr:Al_2_O_3_, Tm:Al_2_O_3_, Lu:Al_2_O_3_, La:Al_2_O_3_, Nd:Al_2_O_3_, Gd:Al_2_O_3_, Yb:Al_2_O_3_ and Dy:Al_2_O_3_ were used to identify the relative population of α- and θ-domains alumina in these samples. Each dopant had a unique effect on alumina, as shown in the plots (Fig. 4). The 2D MQ-MAS spectrum of Pr-doped Al_2_O_3_ (Fig. 4) included three clear transitions at ~59.3, ~13.9 and ~8.8 ppm, respectively. The 2D MQ-MAS of La- and Yb-doped Al_2_O_3_ also showed three transitions, albeit with weaker downfield peaks. The 2D MQ-MAS results for Tm-, Nd-, Lu-, Dy-, Gd- and Yb-doped Al_2_O_3_ also exhibited two upfield peaks at various intensities (Fig. 4c–h). Additionally, the 2D MQ-MAS further highlighted the presence of θ-Al_2_O_3_ in various concentrations as a second minor component for Yb, Gd, La, Nd and Tm-doped Al_2_O_3_. The presence of the dopants in the lattice generated stress in the geometry of arranged alumina, leading to changes in the system configurations. It should be noted that Pr-doped alumina showed a significant increase in θ-domains (~59.3 ppm, Fig. 4b), whereas the Dy and Lu dopants showed no indication of θ-domains (Fig. 4c and i). Since the ratio of Al to Ln was constant, it was assumed that an increase in one type of domain was followed by the decrease of another type of domain by the same amount. Whether or not the dopant favored formation of the θ-domain or α-domain at a given temperature was dictated by the size of the dopant and the size of the site it was being doped into.

**Figure 5:**
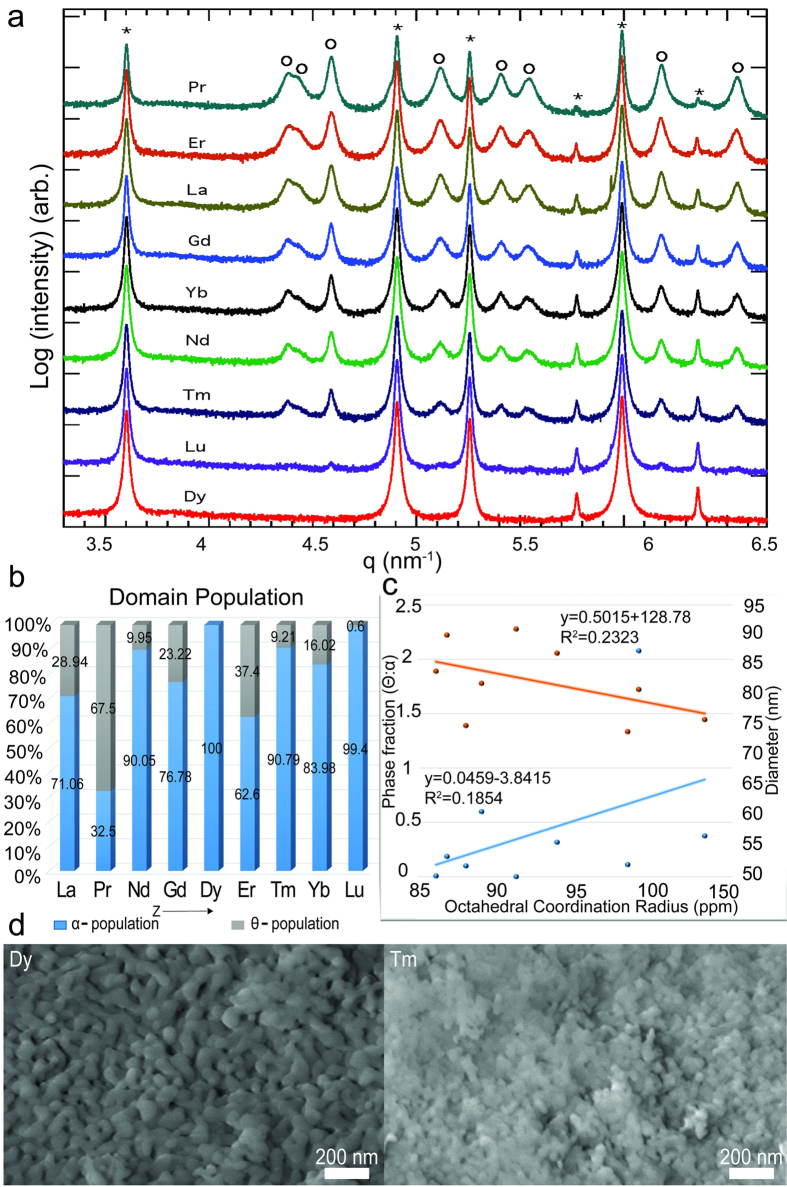
(**a**) Low angle HR-XRD data of the Ln-doped alumina. From the top to the bottom, Ln = Pr, La, Gd, Yb, Nd, Tm, Lu, Dy, respectively. The data were sequentially offset by a factor of 10 for clarity. The peaks denoted by “*” represented the α-phase and the peaks denoted by “o” symbols represented the θ-phase. (**b**) Graphical representation of the α- and θ-phase populations at ~1300 °C. The Dy- and Lu-doped alumina exhibited only α-phase. (**c**) Graphical representation of Ln-cationic phase fraction, octahedral coordination radius and phase diameters. The data points represented Ln-series elements in decreasing order of atomic number, moving from left to right (Lu, Yb, Tm, Er, Dy, Gd, Nd, Pr, and La). The diameter was represented by the orange data points and the phase fraction was represented by the blue data points. A refinement summary of all Ln^3+^ doped alumina was included. (**d**) SEM images of Dy- and Tm-doped alumina.

The final confirmation of the presence of α- and θ-phases in various ratios for each Ln-dopant was determined using data obtained from HR-XRD. This quantitatively reinforced conclusions drawn based on the ss-NMR results for the Ln-doped alumina. HR-XRD spectra of each Ln-dopant indicated notable differences (Fig. 5a) in α- and θ-peaks, with Dy- and Lu-doped alumina exhibiting only α-phase, and Pr-doped alumina revealing the θ-phase in extremely high quantities. It should be noted that all of these doped Al_2_O_3_ samples were prepared at the same calcination temperature of ~1300 °C for 30 minutes. As observed in the case of Er-doped alumina, the transition temperature of α-Al_2_O_3_ increased to ~1325 °C. Therefore, the θ-Al_2_O_3_ domains in the sample prepared at ~1300 °C were expected to be present, since the material was calcined at a temperature lower than the transition temperature for alpha alumina. These spectra indicated the presence of uneven phase percentages in each Ln-doped Al_2_O_3_ sample. The graphical representations of phase populations for each dopant were generated using Rietveld refinement data from the HR-XRD (Fig. 5b), which were analyzed using X-ray refinement software, GSAS-II^39^. They provided a quantitative summary of the phases in each sample doped with Ln-elements. With the exception of Dy- and Lu-doped alumina, the θ-Al_2_O_3_ phase was present in all of the other samples. This suggested that the Dy dopant decreased the α-θ phase transition temperature when compared to the pure alumina phase. The alpha phase transformation temperatures of Dy- and Lu-doped alumina were expected to be significantly lower than ~1300 °C, since a complete transformation of α-phase was observed. Based on this hypothesis, the presence of θ-domains in all other samples clearly suggested that the dopants increased the transition temperatures above ~1300 °C. This temperature requirement was much higher in the case of Pr-doped alumina, as a significantly high population of θ-Al_2_O_3_ domains were observed after calcining at ~1300 °C. The Ln ionic size effect appeared to play a role in the transition temperature shift. There was a notable variation in the phase population of θ- and α- domains (Fig. 5c). It was possible that elements from the second half of the Lanthanide series (Gd, Dy, Tm, Lu, Yb), which were smaller in size due to Lanthanide contraction, could have led to a lower percentage of θ-phase when compared to elements from the first half of the series (La, Pr). The smaller dopant sizes could have resulted in less distortion of the neighboring lattice, leading to a lower energy requirement for atomic rearrangement. Furthermore, the Ln-dopant effects were purely intrinsic, and did not lead to formation of any undesirable LnAlO_3_ or Ln_2_O_3_ phases, which was confirmed by both ss-NMR and HR-XRD spectra. The refinement summary from HR-XRD also provided the geometry and dimensions of the domains, indicating that the ionic size effect of Ln-dopants (Fig. 5c, and Table S1 of Supporting Information) was responsible for delaying the phase transformation of θ-domains into α-domains. The theta alumina particle morphology was anisotropic (ellipsoid-like) whereas the alpha alumina particles were isotropic (cube or sphere-like). The SEM images of two representative Ln-doped alumina samples (Dy and Tm doped alumina, Fig. 5d) were consistent with the structural conclusions from the refinement summary, verifying that contrasting morphologies of the samples were clearly shown. The full SEM images for all Ln-doped alumina samples are shown in the Supporting Information. The Pr-doped alumina sample revealed spherical particles within the elliptical-rich matrix due to significantly large populations of theta alumina, while the Gd-doped alumina sample showed elliptical particles in the spherical-rich matrix, and the Dy- and Lu-doped alumina samples exhibited only spherical particles due to their pure α-phases. Similarly, the other samples were analyzed using SEM, providing rough estimates of their phases and phase populations, which aligned well with the high resolution X-ray diffraction analysis. These dopants changed the basic structure of the host lattice in such a way that the transition temperatures of the polymorphs required more energy to form. With all doped materials being processed under the same furnace conditions (1300 °C for 30 minutes), the materials developed different theta and alpha phase ratios. Since each phase of alumina exhibited a different geometry as dictated by its structure, and the presence of a certain percentage of those phases in the sample resulted in a unique geometry (as shown in the SEM images), it further suggested that not all particles formed alpha alumina during the calcination cycle (i.e. grains were of the theta polymorph). Dopants residing at the grain boundaries were unlikely to generate enough lattice distortions to demonstrate such a notable differences in the phase transformation temperatures. This eliminated scenario (v), leaving scenario (iv) as the only possible location for the Ln-dopants, indicating that they resided in the one-third vacant octahedral sites since the small dimensions of the vacant tetrahedral sites could not accommodate the large-sized Ln^3+^ dopants.

## Conclusions

In summary, Ln doping (400 ppm) in alumina had a significant influence over structural and phase evolution. Ln dopants served as structural promoters to increase the phase transformation temperature (θ → α) by a notable magnitude, which delayed the onset of alumina lattice phase transformation. Ln doping in alumina enabled control of the phase population, with no additional unwanted phases (such as LnAlO_3_ or Ln_2_O_3_) observed. Additionally, it was determined that the Ln dopants resided in the vacant octahedral locations within the alumina lattice. The formation of 100% α-phase was also observed in the case of Dy- and Lu-doped alumina. This study revealed a new perspective on the significance of Ln-doping in alumina.

## Experimental Details

Synthesis of Ln-doped alumina powders was achieved by starting with an *in-situ* nano-precipitation method to synthesize ammonium aluminum hydroxide carbonate (NH_4_Al_1−x_RE_x_(OH)_2_CO_3_) in an aqueous environment. The resulting powder was calcined at 1300 °C for 30 minutes in air. The detailed synthesis method is described in the Supporting Information section. The structural and chemical composition characterization was carried out by using an FEI Quanta 450FEG Scanning Electron Microscope. High resolution synchrotron powder diffraction data were collected using beamline 11-BM at the Advanced Photon Source (APS), Argonne National Laboratory. Discrete detectors covering an angular range from −6 to 16° 2θ were scanned over a 34° 2θ range, with data points collected every 0.001° 2θ at a scan speed of 0.01°/s.

All NMR data was recorded on a Bruker AVIII 400 MHz NMR Spectrometer with a 4 mm, two-channel MAS probe. Approximately 75 mg of material was packed into a 4 mm Zirconia rotor with Kel-F drive cap (Wilmad Lab Glass) and spun at the magic angle using a rate denoted in the text. The magic angle was confirmed using a KBr sample. ^27^Al NMR pulses, offset, and chemical shift references were calibrated and check periodically during the data acquisition run using an alumina (powder) sample. For 1D ^27^Al MAS spectra, the Bruker pulse program, excitation pulse, interscan delay, acquisition time, number of scans, sweep width, and experiment duration were “onepulse”, 0.6 μs at 100 W, 0.5 s, 16 ms, 20480, 600 ppm and 3 h, respectively. The data were processed by zero filling FID to 4096 points, Fourier Transform, phase correction, and referencing. For 2D ^27^Al MQ-MAS spectra, the precise acquisition parameters were optimized for each individual sample. A representative example of the Bruker pulse program, excitation, conversion and selective pulses, interscan delay, acquisition time in F2 and F1, sweep width in F2 and F1, and experiment time were 7 μs at 73 W, 2.5 μs at 73 W, 30 μs at 350 mW, 0.5 s, 24 ms, 10 ms, 400 ppm, the MAS spin rate (10 or 12 kHz, see figure caption) and three days, respectively. The data were processed by zero filling the matrix to 4096 × 512 complex points, 100 Hz line broadening in F2, Fourier Transform in both F2 and F1, phase correction, and shearing transform.

## Additional Information

**How to cite this article:** Patel, K. *et al*. Structural Effects of Lanthanide Dopants on Alumina. *Sci. Rep.* **7**, 39946; doi: 10.1038/srep39946 (2017).

**Publisher's note:** Springer Nature remains neutral with regard to jurisdictional claims in published maps and institutional affiliations.

## Supplementary Material

Supporting Information
