# Supplementary material for: Structural Effects of Lanthanide Dopants on Alumina
Source: Sci Rep. 2017 Jan 6;7:39946. doi: 10.1038/srep39946 (PMC5216414; doi:10.1038/srep39946)
Supplement: Supporting Information [file srep39946-s1.doc]

**Supporting Information**

Structural Effects of Lanthanide Dopants on Alumina

Ketan Patel^†,a^, Victoria Blair^†,b^, Justin Douglas^c^, Qilin Dai^a^, Yaohua Liu^d^, Shenqiang Ren*^,a^, Raymond Brennan*^,b^

1. Department of Mechanical Engineering and Temple Materials Institute, Temple University, Philadelphia, PA 19122
2. U.S. Army Research Laboratory, Aberdeen Proving Ground, MD 21005
3. Molecular Structures Group, University of Kansas, Lawrence, KS 66045
4. Quantum Condensed Matter Division, Oak Ridge National Laboratory, Oak Ridge, TN 37831

* [shenqiang.ren@temple.edu](mailto:shenqiang.ren@temple.edu) and [raymond.e.brennan.civ@mail.mil](mailto:raymond.e.brennan.civ@mail.mil)

**Synthesis Method**

All of the Ln-doped alumina samples discussed in this study were prepared by preparing an acidic solution of aluminum (III) nitrate hydrate, magnesium (II) nitrate hydrate, and lanthanide (III) nitrate hydrate in stoichiometric amounts, resulting in a composition of Ln_0.002_Al_1.998_O_3_ with 250 ppm of MgO. The addition of magnesium to alumina served the dual purpose of creating structural distortions to assist in dissolving Ln into the Al octahedral sites and acting as a grain growth inhibitor. An in-situ nano-precipitation method was used to synthesize ammonium aluminum hydroxide carbonate (NH_4_Al_1-x_RE_x_(OH)_2_CO_3_) powder in an aqueous environment. Solution B, a basic solution, consisted of 11% by weight of ammonium bicarbonate and 3% by weight of ammonium hydroxide in DI water.

Once solutions A and B were prepared, a third solution, referred to as Buffer, consisted of 2% by weight ammonium bicarbonate in DI water and was where the reaction would take place. The pH of the Buffer was adjusted to ~7 by adding a small amount of nitric acid. Finally, solutions A and B were added drop-wise to the Buffer solution in such a way that the pH remained at ~7 during the entire precipitation exercise. When solution A was exhausted, the resulting suspension was allowed to age while stirring vigorously overnight. The next day, the suspension was filtered from the remnant salt solution. The resulting powder was washed twice with DI water and once with isopropyl alcohol. After washing, the powder was put into an oven to dry. The dry powder was gently crushed and calcined at 1300°C for 30 minutes at a heating rate of 10°C/min.

A variety of Ln-doped samples (La, Pr, Nd, Gd, Dy, Tm, Yb, and Lu- doped alumina) were prepared to observe the dopant effects on the alumina nanocrystals. Figures S1a, S1b and S1c represent Pr-doped, Dy-doped and Gd-doped alumina, respectively, in which the contrasting morphologies of the samples are clearly shown. It was quite interesting to see the morphology changes, which were a consequence of the Ln^3+^ dopants, since the Ln-dopants shared the same oxidation state as aluminum.

High resolution solid-state nuclear magnetic resonance (ss-NMR) spectroscopy was used to investigate the effects of Ln-ions on the phase composition of the alumina matrix. Due to differences in the anisotropic electron density surrounding the Al nuclei sites in different phases of Al_2_O_3_, the ^27^Al chemical shift provided an accurate and precise readout of the material phase. Furthermore, 1D magic angle spinning (MAS) and 2D ^27^Al multiple quantum magic angle spinning (MQ-MAS) spectra were employed to measure phase compositions of doped Al_2_O_3_. In addition, high resolution X-ray diffraction techniques were implemented to study the phase composition and transformation under different lanthanide cation dopants.

**
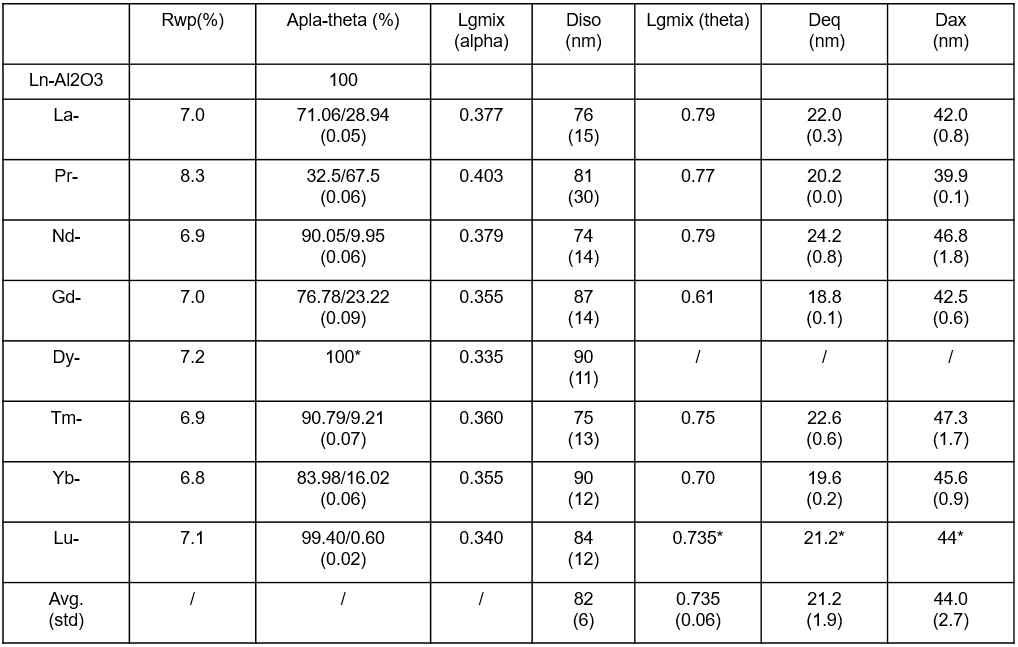
**

**Table S1.** Refinement summary of all Ln^3+^ doped alumina.

**
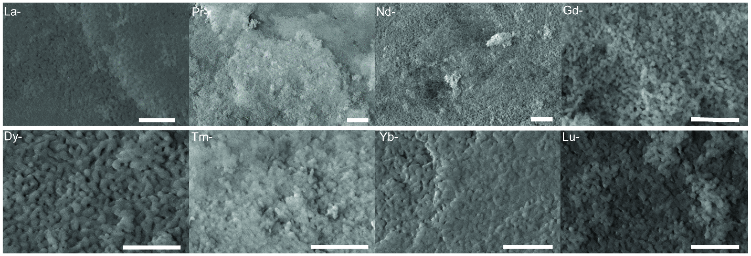
**

**Figure S1.** SEM images of Ln-doped alumina. The scale bar is one micron.


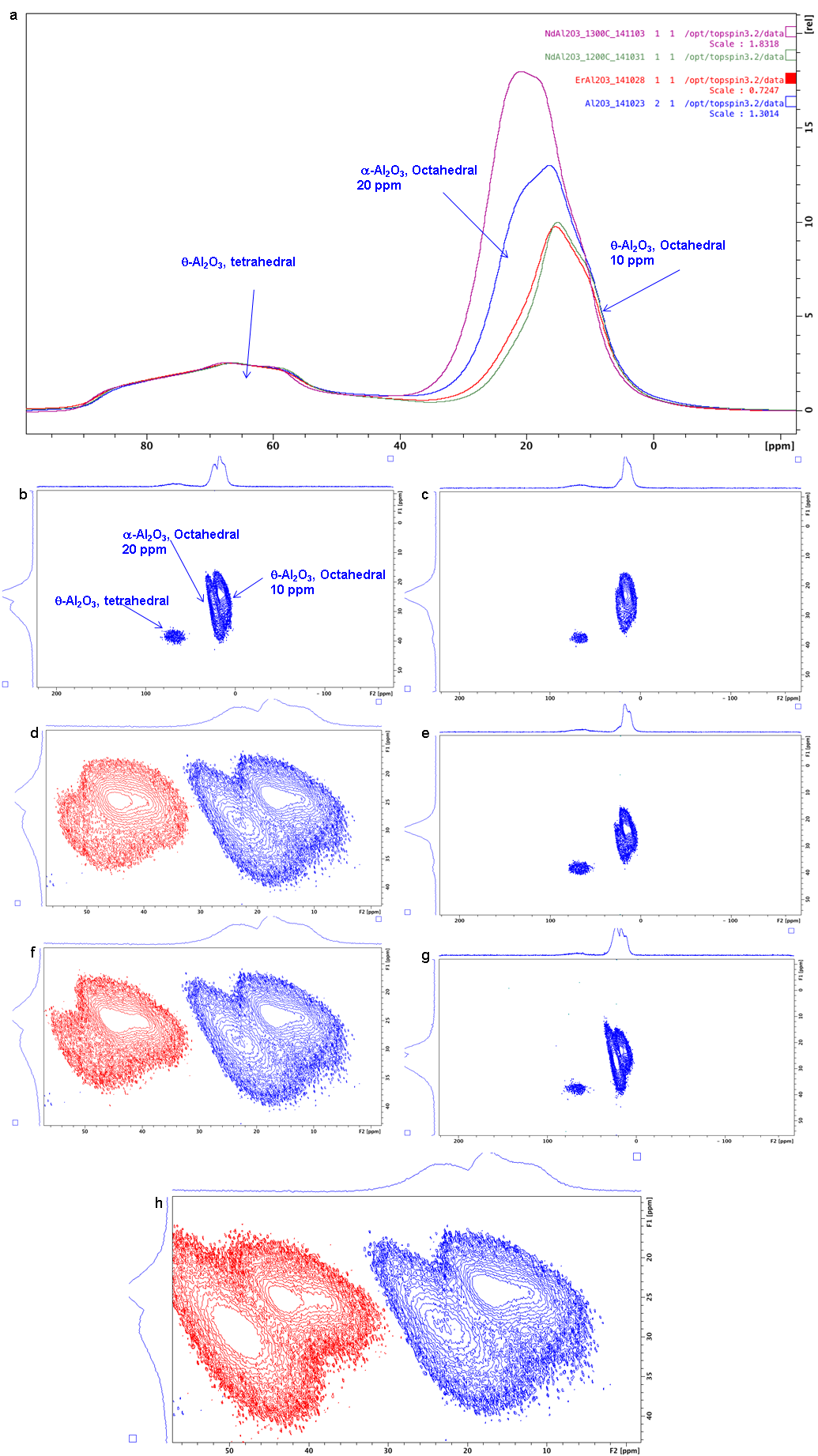


**Figure S2.** (a) Overlay of 1D MAS spectra of Al_2_O_3_ (blue), Er-doped Al_2_O_3_ (red), Nd-doped Al_2_O_3_ sintered at 1200°C (green), and Nd-doped Al_2_O_3_ at sintered 1300°C (purple). (b) 2D mapping of Al_2_O_3_ (c) 2D mapping of Er-doped Al_2_O_3_ (d) 2D mapping of Al_2_O_3_ (blue) and Er-doped Al_2_O_3_ (red), which has been offset to left for clarity (e) 2D mapping of Nd-doped Al_2_O_3_ (1200°C sintered). (f) 2D mapping of Al_2_O_3_ (blue) and Nd-doped Al_2_O_3_ (1200°C sintered, red), which has been offset for clarity. (g) 2D mapping of Nd-doped Al_2_O_3_ (1300°C sintered). (h) 2D mapping of Al_2_O_3_ (blue) and Nd-doped Al_2_O_3_ (sintered at 1300 °C, red), which has been offset for clarity.


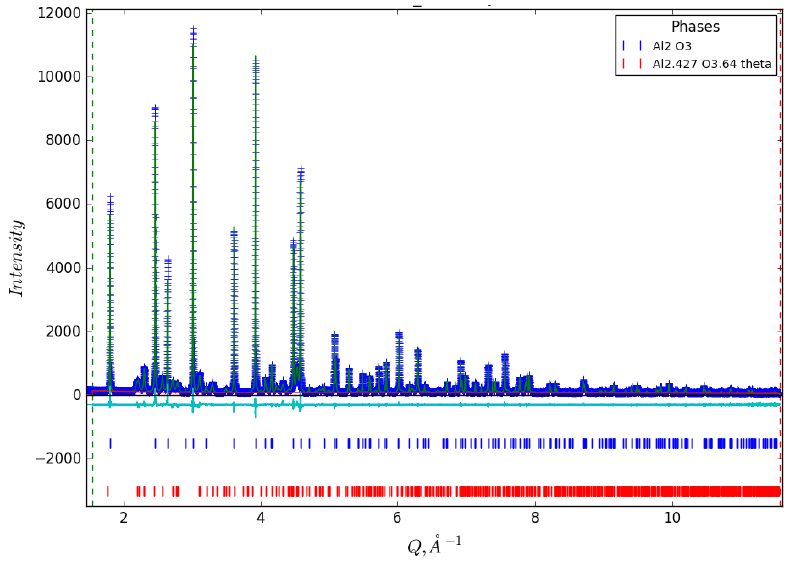


**Figure S3**. Rietveld refinement of Er-doped Al_2_O_3_.

**Figure S4**. Rietveld refinement of Dy-doped Al_2_O_3_.

**Figure S5**. Rietveld refinement of Lu-doped Al_2_O_3_.

**Figure S6**. Rietveld refinement of Tm-doped Al_2_O_3_.

**Figure S7**. Rietveld refinement of Nd-doped Al_2_O_3_.

**Figure S8**. Rietveld refinement of Yb-doped Al_2_O_3_.

**Figure S9**. Rietveld refinement of Gd-doped Al_2_O_3_.

**Figure S10**. Rietveld refinement of La-doped Al_2_O_3_.

**Figure S11**. Rietveld refinement of Pr-doped Al_2_O_3_.
